# The Book World of Medicine and Science

**Published:** 1897-02-27

**Authors:** 


### THE BOOK WORLD OF MEDICINE AND SCIENCE.

A COURSE OF ELEMENTARY PRACTICAL PHYSIOLOGY AND HISTOLOGY. By M. FOSTER, M.D., F.R.S., Professor of Physiology in the University of Cambridge, and J. N. LANGLEY, M.A., F.R.S., Fellow of Trinity College, Cambridge. (Pp. 413, with 10 figures. Macmillan and Co. 1896. Price 6s.)

This well-known text-book has now been before the public for twenty years, and that it can still hold the field against its numerous younger rivals is shown by the frequency with which it has of late been reprinted. One of the factors in its success is undoubtedly its completeness; within its comparatively modest compass it includes the elements of histology, of experimental physiology, and of physiological chemistry. The scope of the work is essentially practical, the greatest possible care being always devoted to the exact description of the methods recommended. In this connection it is most satisfactory to note that the experiments to be performed by the demonstrator are simply mentioned without description, whereby the student is spared the annoyance of wading through a large amount of tedious and unnecessary detail. The obvious intention of the authors has been that the book should be used in a practical class held in connection with a theoretical course of animal physiology. The arrangement of the chapters hence follows a convenient order for the study of the various organs and tissues, and does not depend upon the chemical, experimental, or histological nature of their subject-matter. On casual inspection the sequence may, as a result, appear somewhat haphazard, but regarded from the correct point of view, that of an adjunct to theoretical study, its educational value is much greater than if its contents had been grouped under more formal headings. If there is one fault which we feel inclined to impute in some measure to Professor Foster and Mr. Langley it is a tendency to rest a little too much upon their well-earned laurels. The former is himself credited with the remark that no physiological fact is worth three years' purchase, and although this does not, of course, apply equally to methods, it is none the less worthy of comment that the book under notice has only changed during the last twelve years by the addition of less than twenty pages. This period has been one of great technical advance, and we must confess to a feeling of surprise at finding no mention whatsoever of Golgi's method of staining the central nervous system, a method which, in whatever sense one may interpret its results, has undoubtedly opened up a new chapter in neurology. On the other hand, the book is singularly free from faults of commission; it is excellently got up (save for an ugly misprint on p. 394), and can be cordially recommended as the most compact, lucid, and reliable work of its kind in the English language.

THE NATURAL AND ARTIFICIAL METHODS OF FEEDING INFANTS AND YOUNG CHILDREN. By EDMUND CAUTLEY, M.D. 8vo., 364 pp. (London: J. and A. Churchill. 1897. Price 7s. 6d.)

The author of this book aims at giving in a handy form the advances which have recently been made in the methods of infant feeeding as based on the study of physiological chemistry. Much has been done of late years to make the foundation of dietetics more secure, but it must be confessed that, in the case of children at least, some of the most elementary rules are almost more honoured in the breach than in the observance. That this is due to lack of exact knowledge of the subject there can be little doubt, and we hope the volume before us will aid in removing the reproach. Dr. Cautley approaches this subject from the scientific side, and provides a mass of data not before readily accessible in this country. The composition of both human and cows' milk, and the alteration it undergoes under various conditions, are discussed in detail, and numerous analyses are given, tables being very freely used throughout the book; not that the clinical aspects of the subject are neglected, as both breast and artificial feeding are very fully dealt with, but the fact that chemical and physiological knowledge is always used as a basis undoubtedly adds to the value of the book as a work of reference. The preparation of cows' milk to approximate it to the natural food of the infant receives careful attention, and formulæ for numerous milk mixtures suitable for different ages are given, the chemical composition being appended to each. Proprietary foods do not find much favour with the author, but he gives analyses of those in most frequent use, which should prove useful to those who employ them. The dietary of premature infants and of older children is also discussed, and the book concludes with an excellent chapter on the growth of children. The book is an admirable one, and if the numerous tables occasionally interrupt the continuity of the text it is at least well that our practice in the feeding of infants should be according to reason and scientific teaching rather than rest on the insecure foundations of empiricism or experiment.

THE COTTAGE HOSPITAL CASE BOOK, OR REGISTER OF PATIENTS. (London: The Scientific Press.)

This is a book which will be found of great service by the officials of cottage hospitals and small provincial hospitals. There can be no doubt as to the want which exists for such a book. No sooner does a new hospital get to work than the necessity for some form of record of the cases treated within it becomes apparent. A book is devised on the spur of the moment, and by many steps and devious processes of evolution it is gradually improved; but however great the improvement in individual cases, the end is that every hospital has its own style, that there is no attempt at uniformity, and that comparison between hospital and hospital is a matter of the greatest difficulty.

The object of the publishers of this case book has been to provide a means of displaying at once all the principal details regarding the cases concerned. Each opening, *i.e.*, each double page, contains rulings for six patients, plenty of space being provided for entering the name, age, condition, disease, subscriber giving recommendation, the amounts of the various payments made, the dates on which they are received, the result of the case, and the date at which it is discharged.

For each case rulings are provided for eight weekly payments, which may be taken to cover the average stay of patients in cottage hospitals, and is, in fact, considerably longer than most "letters" are allowed to remain in force.

The book is made in two sizes—one for hospitals treating not more than 150 in-patients in the year, the other for larger hospitals treating not more than 300 in-patients per annum. There is no cramping or overcrowding of details on the pages, the provision of eight lines for cash entries for each patient having compelled the provision of plenty of space for all other details.

There can be no doubt of the great advantage of the use of such a register, not merely for the sake of facility of reference or comparison afterwards, but because, by the smallest possible number of entries, the exact position of each patient is displayed at a glance.

